# Composition of gut and oropharynx bacterial communities in *Rattus norvegicus* and *Suncus murinus* in China

**DOI:** 10.1186/s12917-020-02619-6

**Published:** 2020-10-31

**Authors:** Wen-qiao He, Yi-quan Xiong, Jing Ge, Yan-xia Chen, Xue-jiao Chen, Xue-shan Zhong, Ze-jin Ou, Yu-han Gao, Ming-ji Cheng, Yun Mo, Yu-qi Wen, Min Qiu, Shu-ting Huo, Shao-wei Chen, Xue-yan Zheng, Huan He, Yong-zhi Li, Fang-fei You, Min-yi Zhang, Qing Chen

**Affiliations:** 1 Department of Epidemiology, School of Public Health, Guangdong Provincial Key Laboratory of Tropical Disease Research, Southern Medical University, 1838 Guangzhou North Road, Guangzhou, 510515 China; 2 Chinese Evidence-based Medicine Center and CREAT Group, West China Hospital, Sichuan University, Chengdu, 610000 China; 3 Medical Office of Wuxi People’s Hospital, Wu Xi, 214000 China

**Keywords:** *Rattus norvegicus*, *Suncus murinus*, Bacterial composition, Next-generation sequencing

## Abstract

**Background:**

*Rattus norvegicus* and *Suncus murinus* are important reservoirs of zoonotic bacterial diseases. An understanding of the composition of gut and oropharynx bacteria in these animals is important for monitoring and preventing such diseases. We therefore examined gut and oropharynx bacterial composition in these animals in China.

**Results:**

Proteobacteria, Firmicutes and Bacteroidetes were the most abundant phyla in faecal and throat swab samples of both animals. However, the composition of the bacterial community differed significantly between sample types and animal species. Firmicutes exhibited the highest relative abundance in throat swab samples of *R. norvegicus*, followed by Proteobacteria and Bacteroidetes. In throat swab specimens of *S. murinus*, Proteobacteria was the predominant phylum, followed by Firmicutes and Bacteroidetes. Firmicutes showed the highest relative abundance in faecal specimens of *R. norvegicus*, followed by Bacteroidetes and Proteobacteria. Firmicutes and Proteobacteria had almost equal abundance in faecal specimens of *S. murinus*, with Bacteroidetes accounting for only 3.07%. The family Streptococcaceae was most common in throat swab samples of *R. norvegicus*, while Prevotellaceae was most common in its faecal samples. Pseudomonadaceae was the predominant family in throat swab samples of *S. murinus*, while Enterobacteriaceae was most common in faecal samples. We annotated 33.28% sequences from faecal samples of *S. murinus* as potential human pathogenic bacteria, approximately 3.06-fold those in *R. norvegicus*. Potential pathogenic bacteria annotated in throat swab samples of *S. murinus* were 1.35-fold those in *R. norvegicus*.

**Conclusions:**

Bacterial composition of throat swabs and faecal samples from *R. norvegicus* differed from those of *S. murinus*. Both species carried various pathogenic bacteria, therefore both should be closely monitored in the future, especially for *S. murinus*.

**Supplementary information:**

**Supplementary information** accompanies this paper at 10.1186/s12917-020-02619-6.

## Background

Approximately 61% of human pathogens are of animal origin, with some commensal wild animals being important reservoirs for a wide range of zoonotic pathogens [1]. *Rattus norvegicus* (Norway rat) is one of the dominant commensal rodents in urban environments worldwide [2]. However, with rapid and continuous urbanization, *Suncus murinus* (Asian house shrew), once a non-commensal species, is becoming widespread and adapting to urban environments in Asia and eastern Africa [3]. Due to their many opportunities for close contact with humans, these two animals are considered to pose a risk to human health [2, 3].

A large number of bacterial species live in the oropharyngeal and gastrointestinal tracts of mammals, which evolve unique bacterial communities. Under certain conditions, some bacterial species carried by animals can be transmitted to humans and cause infections, such as through animal bites, faeces, airborne saliva droplets, or ectoparasitic arthropod vectors. For instance, *Salmonella* from rodents can cause food-borne diseases in humans through faecal contamination of water and foods [4]. *Streptobacillus moniliformis* and *Spirillum minus* infect humans through rodent bites or scratches, or mucocutaneous contact with animals’ saliva, urine, or faeces [5]. *Bacillus anthracis* can be transmitted from infected rodents to humans through cutaneous, gastrointestinal, or inhalation routes [6]. Over the past decade, methicillin-resistant *Staphylococcus aureus* (MRSA), an emerging drug-resistant bacterium, has been found in the oropharynx and rectum of *R. norvegicus* and the oropharynx of *S. murinus* [7, 8]. Therefore, an understanding of the composition of the bacterial communities in the gut and oropharynx of *R. norvegicus* and *S. murinus* is important for monitoring and preventing rodent-borne bacterial diseases.

Next-generation sequencing (NGS) is a highly efficient approach for the detection of microbiota. Over the last decade, NGS-based 16S rRNA gene amplicon sequencing has been used in a wide variety of fields, such as revealing the bacterial community composition of environments and animals and investigating the relationship between microbiota and diseases. Several studies have used the NGS-based 16S rRNA gene amplicon sequencing method to investigate the composition of bacterial communities in rats and *S. murinus*. A comparison of the gut bacterial communities between laboratory and wild *S. murinus* revealed a higher microbial diversity in the latter [9]. Other studies have revealed the bacterial communities of the gastrointestinal and respiratory tracts of laboratory rats [10]. However, to the best of our knowledge, there is no literature addressing the composition of the oropharynx bacterial communities of *S. murinus* or *R. norvegicus*. In addition, the composition of the bacterial community of the gastrointestinal tract in wild *R. norvegicus* remains unknown.

To elucidate gut and oropharynx bacterial composition in *R. norvegicus* and *S. murinus*, we surveyed throat swab and faecal samples of these animals using NGS-based 16S rRNA gene sequencing.

## Results

### Samples

We trapped 643 wild *R. norvegicus* and 313 wild *S. murinus*. Throat swab and faecal samples from 12 individuals of each species were randomly selected for NGS-based 16S rRNA gene sequencing. The results from the selected samples were divided into four groups based on animal species and type of samples (faecal samples from *R. norvegicus*; faecal samples from *S. murinus*; throat swab samples from *R. norvegicus*; throat swab samples from *S. murinus*; Table S1).

### Data overview

An average of 87,022 raw reads, 81,990 clean tags and 78,666 effective tags was obtained for each sample (Table S1). The raw data have been submitted to the NCBI Sequence Read Archive (SRA) dataset (SRR11449241-SRR11449288). The mean length of each effective tag was 253 bp. After removing operational taxonomic units (OTUs) represented by fewer than 20 sequences, we identified 2959 OTUs with an average of 823 OTUs for each sample (Table S1). Rarefaction curves tended to be flat when the number of detected sequences reached about 50,000 (Figure S1). In addition, Good’s coverage indices were very high (99.1–99.7%), indicating that the depth of sequencing was sufficient to reflect the composition of the bacterial community of the samples.

**Fig. 1:**
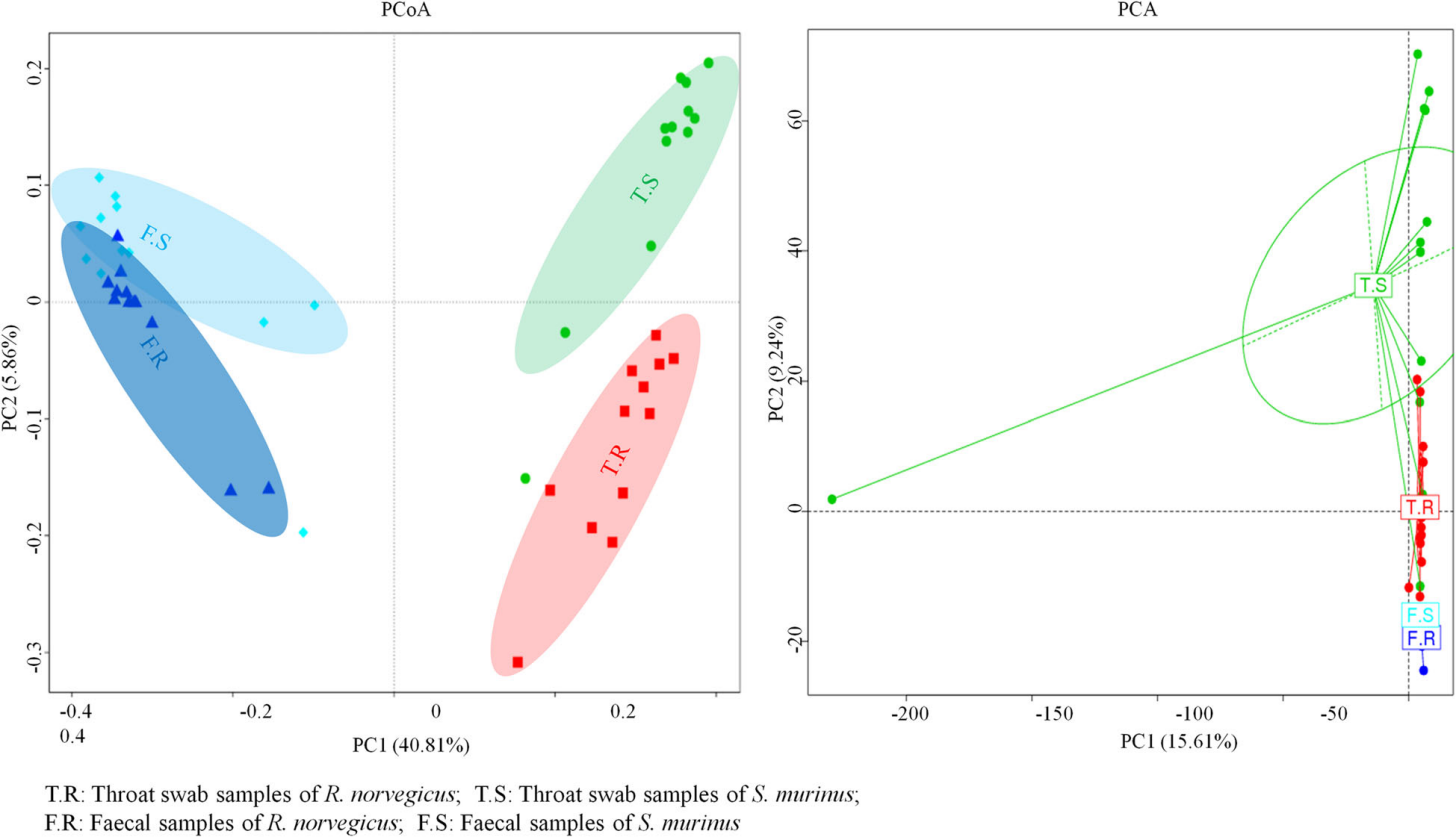
Principal component analysis (PCA) and principal coordinates analysis (PCoA) plots (unweighted UniFrac distances) between different samples. Each sign represents an individual sample. The sample groups were clearly separated. These data indicated that the microbial communities were different in different animals and different sample types

**Fig. 2:**
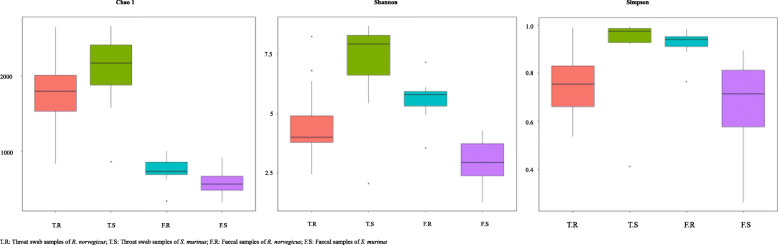
Chao 1, Shannon and Simpson indices of different groups of samples. The throat swab samples had a significantly greater number of microbial species than the faecal samples. The bacterial richness and evenness in throat swab samples of *S. murinus* was higher than that of *R. norvegicus*, while the bacterial richness and evenness in faecal samples of *R. norvegicus* was higher than that of *S. murinus*

Principal component analysis (PCA) and principal coordinates analysis (PCoA) demonstrated the distinctive composition of the bacterial community between the four groups (Fig. 1). ANOSIM analysis also confirmed that there was significant separation between the different groups (Table 1). There were significantly greater numbers of bacterial species in throat swab samples than faecal samples (Fig. 2). Bacterial richness and evenness were higher in throat swab samples of *S. murinus* than in those of *R. norvegicus*, while bacterial richness and evenness was greater in faecal samples of *R. norvegicus* than in those of *S. murinus* (Fig. 2).

**Table 1:** ANOSIM analysis for different groups of samples

Group	F.R-T.R	F.S-T.R	F.S-F.R	T.S-T.R	T.S-F.R	T.S-F.S
*R*-value	0.9952	0.99	0.5736	0.8347	0.9862	0.9059
*P*-value	0.001	0.001	0.001	0.001	0.001	0.001

*T.S* Throat swab samples of *Suncus murinus*, *T.R* Throat swab samples of *Rattus norvegicus*, *F.S* Faecal samples of *Suncus murinus*, *F.R* Faecal samples of *Rattus norvegicus*

### Bacterial community composition in throat swab samples

**Fig. 3:**
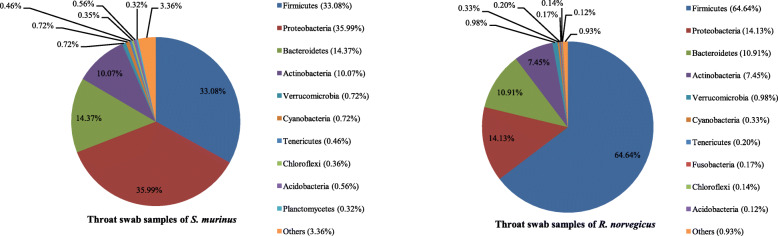
Bacterial community composition in throat swab samples at the phylum level. A total of 41 and 53 phyla were found in the throat swab samples of *R. norvegicus* and *S. murinus*, respectively. The top ten phyla are shown

**Fig. 4:**
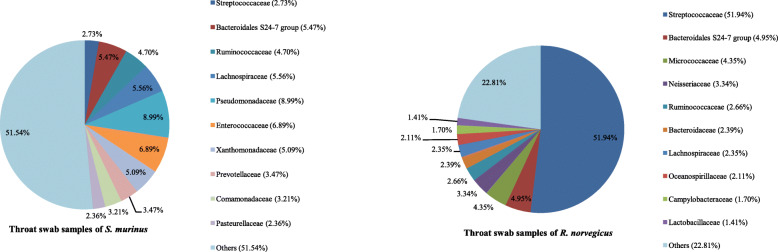
Bacterial community composition in throat swab samples at the family level. A total of 348 and 367 families were found in the throat swab samples of *R. norvegicus* and *S. murinus*, respectively. The top ten families are shown

**Fig. 5:**
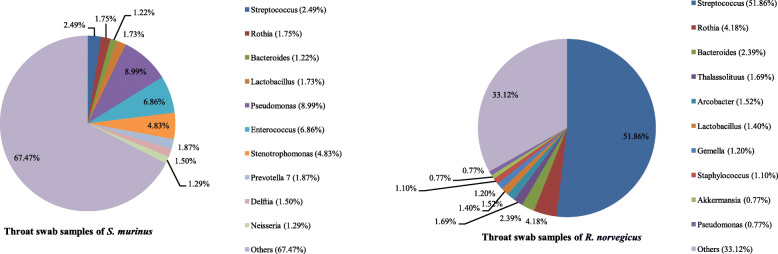
Bacterial community composition in throat swab samples at the genus level. A total of 765 and 891 genera were found in the throat swab samples of *R. norvegicus* and *S. murinus*, respectively. The top ten genera are shown

Sequences from *R. norvegicus* revealed 41 phyla, 105 classes, 179 orders, 348 families and 765 genera of archaea and bacteria, while those from *S. murinus* represented 53 phyla, 121 classes, 194 orders, 367 families and 891 genera. The 10 most abundant phyla, families and genera in throat swab samples of these two animals are shown in Figs. 3, 4 and 5.

The three most abundant phyla in *R. norvegicus* were Firmicutes (64.64% relative abundance) followed by Proteobacteria (14.13%) and Bacteroidetes (10.91%). These three phyla accounted for over 89% of the total sequences (Fig. 3). Streptococcaceae and Streptococcus were the predominant family and genus within the Firmicutes, respectively (Figs. 4 and 5). Neisseriaceae and Thalassolituus were the most common family and genus within the Proteobacteria, respectively. Bacteroidales S24–7 group and Bacteroides were the most common family and genus within the Bacteroidetes, respectively.

Different from *R. norvegicus*, the most common phylum in *S. murinus* was Proteobacteria, which accounted for 35.99% of the total sequences, followed by Firmicutes (33.08%) and Bacteroidetes (14.37%; Fig. 3). Pseudomonadaceae and Pseudomonas were the most common family and genus within the Proteobacteria, respectively (Figs. 4 and 5). Enterococcaceae and Enterococcus were the most common family and genus within the Firmicutes (Figs. 4 and 5). Similar to *R. norvegicus*, Bacteroidales S24–7 group was also the most common family within the Bacteroidetes in *S. murinus* (Fig. 4). However, Prevotella 7 was the most common genus within the Bacteroidetes (Fig. 5). Actinobacteria also had a high relative abundance in the throat swabs of *R. norvegicus* and *S. murinus*, ranking fourth among phyla and accounting for 7.45 and 10.07% of all sequences, respectively.

### Bacterial community composition in faecal samples

**Fig. 6:**
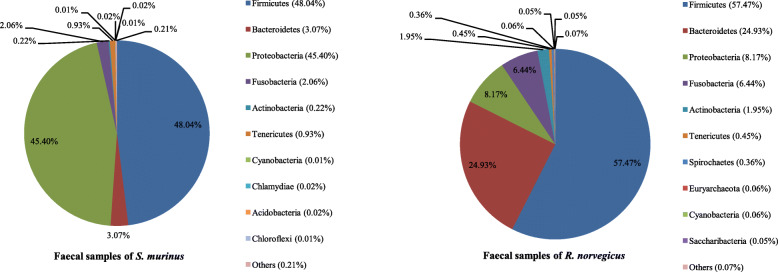
Bacterial community composition in faecal samples at the phylum level. A total of 25 and 31 phyla were found in the faecal samples of *R. norvegicus* and *S. murinus*, respectively. The top ten phyla are shown

**Fig. 7:**
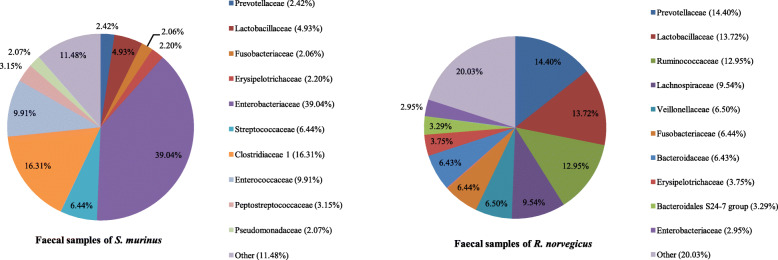
Bacterial community composition in faecal samples at the family level. A total of 156 and 198 families were found in the faecal samples of *R. norvegicus* and *S. murinus*, respectively. The top ten families are shown

**Fig. 8:**
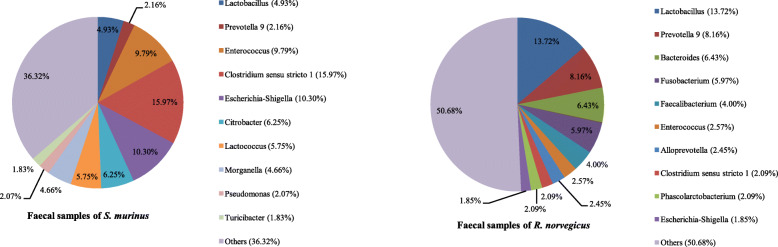
Bacterial community composition in faecal samples at the genus level. A total of 342 and 413 genera were found in the faecal samples of *R. norvegicus* and *S. murinus*, respectively. The top ten genera are shown

Sequences from *R. norvegicus* annotated 25 phyla, 56 classes, 85 orders, 156 families and 342 genera of archaea and bacteria, while those from *S. murinus* represented 31 phyla, 63 classes, 98 orders, 198 families and 413 genera. The 10 most abundant phyla, families and genera in faecal samples are shown in Figs. 6, 7 and 8.

Firmicutes, Bacteroidetes and Proteobacteria were also the three most abundant phyla in faecal samples of *R. norvegicus* and *S. murinus*, whereas Firmicutes was the most common phylum in faecal samples of both animals, accounting for 57.47 and 48.04% of sequences, respectively. Bacteroidetes was the second most common phylum in *R. norvegicus*, while Proteobacteria was the second most common phylum in *S. murinus* (Fig. 6).

For *R. norvegicus*, Lactobacillaceae and Lactobacillus were the most common family and genus within the Firmicutes (Figs. 7 and 8). However, for *S. murinus*, Clostridiaceae 1 and Clostridium sensu stricto 1 were the most common family and genus within the Firmicutes (Figs. 7 and 8). Prevotellaceae and Prevotella 9 were the dominant family and genus within the Bacteroidetes in both animals (Figs. 7 and 8). Enterobacteriaceae was the most common family within the Proteobacteria in both animals, and Escherichia-Shigella was the most common genera within the Enterobacteriaceae (Figs. 7 and 8).

### Comparison of differentially enriched taxa between different groups

**Fig. 9:**
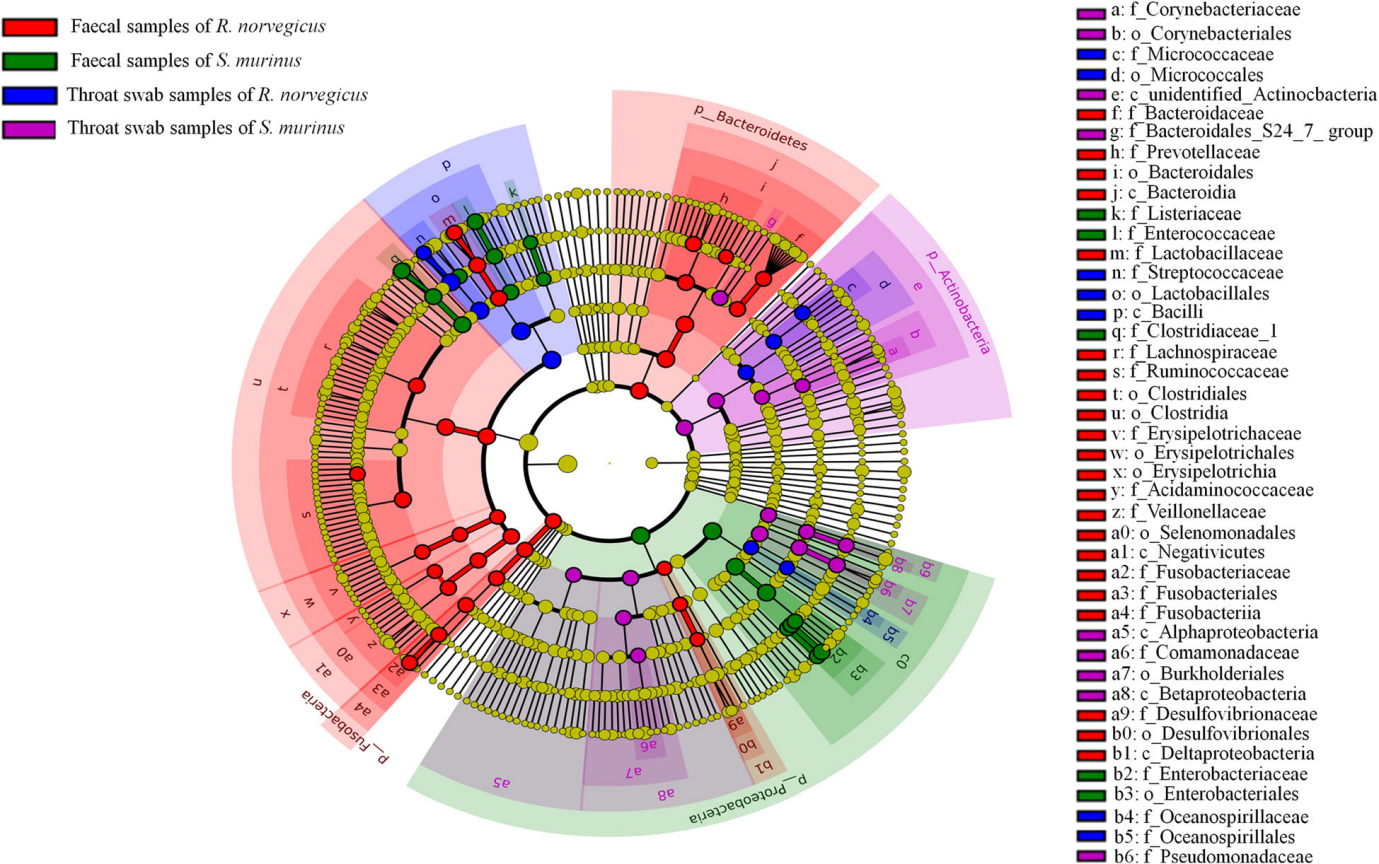
Cladogram plotted from LEfSe analysis showing the taxonomic levels represented by rings with phyla in the outermost ring and genera in the innermost ring. Each circle is a member within that level. Those taxa in each level are coloured by groups for which it is more abundant (*P* < .05)

Figure 9 shows the taxa that were relatively enriched in one group of samples by comparing the four different groups. The families Streptococcaceae (51.94%) within the Firmicutes and Micrococcaceae (4.35%) within the Actinobacteria had a higher relative abundance in throat swab samples of *R. norvegicus* than that in other groups. The families Pseudomonadaceae (8.99%) and Comamonadaceae (3.21%) within the Proteobacteria, Corynebacteriaceae (2.27%) within the Actinobacteria and the Bacteroidales S24–7 group (5.47%) within the Bacteroidetes were significantly enriched in throat swab samples of *S. murinus*.

The families Prevotellaceae (14.40%) and Bacteroidaceae (6.43%) within the Bacteroidetes were enriched in faecal samples of *R. norvegicus* compared to the other three groups. Some families within the Firmicutes were also enriched in faecal samples of *R. norvegicus*, including Lactobacillaceae (13.72%), Ruminococcaceae (12.95%), Lachnospiraceae (9.54%), Veillonellaceae (6.50%), Erysipelotrichaceae (3.75%) and Acidaminococcaceae (2.24%). The families Fusobacteriaceae (6.44%) within the Fusobacteria and Desulfovibrionaceae (2.28%) within the Proteobacteria were also enriched in faecal samples of *R. norvegicus* compared to the other groups.

Enterobacteriaceae (39.04%), Clostridiaceae 1 (16.31%), Enterococcaceae (9.91%) and Listeriaceae (1.47%) were the four families most differentially enriched in faecal samples of *S. murinus* compared to the other groups. Enterobacteriaceae falls within the phylum Proteobacteria, while the other three families belong to the phylum Firmicutes.

### Potential human pathogenic bacteria

Sequences from the four groups of samples were annotated in 21 families of potential human pathogenic bacteria. The top five potential pathogenic families for each group are shown in Table 2. We identified 27 genera of potential human pathogenic bacteria in throat swab samples of the two animals. Faecal samples from the two animals contained sequences annotating 29 genera of potential human pathogenic bacteria.

**Table 2:** Top five potential human pathogenic bacterial families in different groups of samples

	Throat swab samples of *S. murinus*	Throat swab samples of *R. norvegicus*	Faecal samples of *S. murinus*	Faecal samples of *R. norvegicus*
1	*Pseudomonadaceae*	*Streptococcaceae*	*Enterobacteriaceae*	*Bacteroidaceae*
2	*Streptococcaceae*	*Micrococcaceae*	*Streptococcaceae*	*Enterobacteriaceae*
3	*Corynebacteriaceae*	*Neisseriaceae*	*Pseudomonadaceae*	*Streptococcaceae*
4	*Micrococcaceae*	*Bacteroidaceae*	*Listeriaceae*	*Pseudomonadaceae*
5	*Pasteurellaceae*	*Pseudomonadaceae*	*Mycoplasmataceae*	*Spirochaetaceae*

**Fig. 10:**
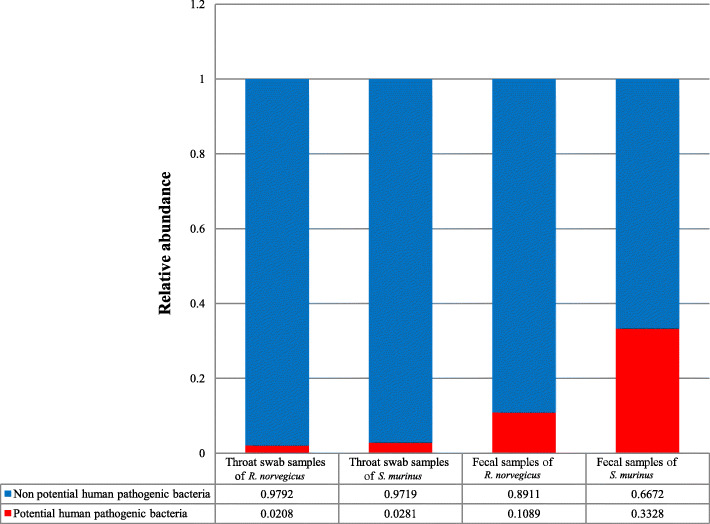
Relative abundance of potential human pathogenic bacteria in each group of samples

A total of 23 species were annotated as potential human pathogenic bacteria in throat swab samples of *R. norvegicus*, while 25 species were found in throat swab samples of *S. murinus*. For faecal samples, we detected 21 and 20 species of potential human pathogenic bacteria in *R. norvegicus* and *S. murinus*, respectively. We found higher relative abundance of potential human pathogenic bacteria in faecal and throat swab samples from *S. murinus* than that in those from *R. norvegicus*. About 33.28% of sequences from faecal samples of *S. murinus* were annotated as potential human pathogenic bacteria, which was approximately 3.06-fold that in *R. norvegicus*. The relative abundance of potential pathogenic bacteria in throat swab samples from *S. murinus* was 1.35-fold that in *R. norvegicus* (Fig. 10).

*Enterococcus durans*, *Escherichia albertii*, *Clostridium perfringens*, *Citrobacter freundii*, *Lactococcus garvieae*, *Delftia tsuruhatensis*, *Clostridium baratii*, *Pseudomonas aeruginosa*, *Sphingobacterium multivorum* and *Comamonas testosteron* were found in all four groups. However, we detected a higher relative abundance of these pathogens in *S. murinus* than in *R. norvegicus*. For instance, *E. albertii* and *C. perfringens* had a relative abundance of over 10% in faecal samples of *S. murinus*. The highest relative abundances of *Helicobacter mastomyrinus*, *Fusobacterium russii*, *Desulfovibrio desulfuricans*, *Prevotella denticola*, *Rothia dentocariosa* and *Prevotella melaninogenica* were in throat swab samples from *S. murinus*. Some opportunistic pathogens, such as *Prevotella loescheii* and *Mycoplasma pneumoniae*, were only found in throat swab samples from *S. murinus*.

*Streptococcus minor*, *Staphylococcus aureus*, *Corynebacterium riegelii*, *Enterococcus cecorum*, *Corynebacterium riegelii* and *Kocuria kristinae* were most common in throat swab samples from *R. norvegicus* among the four groups. To the best of our knowledge, and with the exception of *S. aureus*, these bacteria have not been reported previously in wild rats. The highest relative abundance of *Fusobacterium mortiferum* was found in faecal samples. We did not identify Bartonella, Leptospira, *Streptobacillus moniliformis*, *Spirillum minus,* or *Yersinia pestis* in faecal or throat swab samples.

## Discussion

To the best of our knowledge, this is the first study to reveal and compare the composition of bacterial communities in the oropharyngeal and gastrointestinal tracts of *R. norvegicus* and *S. murinus*. More bacterial species were identified in throat swab and faecal samples of *S. murinus* than in those of *R. norvegicus*. The bacterial communities of these two animals were significantly different, even though they were captured from the same habitats (Fig. 1). A previous study reports that animals of the same species from different habitats harbour similar bacteria [11]. These results indicate that the microbiota have host tropism. Variation was found in the α and β diversity analyses within the sample groups, this might be explained by the different sampling time, body size and sex of the selected animals (Figs. 1 and 2). The difference between the groups was greater than the difference within groups (Table 1).

In human oropharyngeal samples, Firmicutes is the most common phylum, with Streptococcaceae within the Firmicutes being the most common family [12]. We found that Firmicutes and Streptococcaceae were also the most common phylum and family, respectively, in oropharyngeal samples from *R. norvegicus*. However, Proteobacteria was the predominant phylum in oropharyngeal samples of *S. murinus*, with Pseudomonadaceae the most common family (Figs. 3 and 4). This suggests a greater similarity in oropharynx bacterial composition between *R. norvegicus* and humans than between *S. murinus* and humans.

The three most abundant phyla (Firmicutes, Bacteroidetes and Proteobacteria) and predominant genus (Lactobacillus) in faecal samples of *R. norvegicus* in our study were in line with previous results from Sprague–Dawley rats (laboratory rats of the species *R. norvegicus domestica)* (Fig. 6) [13]. This suggests that the bacterial composition of wild and laboratory *R. norvegicus* share a degree of similarity. This phenomenon can also be explained by the host tropism of microbiota.

Firmicutes and Proteobacteria were the dominant phyla in faecal samples of *S. murinus*, accounting for over 90% of the sequences (Fig. 6). This was consistent with the results of a previous study [9]. We only detected a low relative abundance (3.07%) of Bacteroidetes in faecal samples from *S. murinus*. A previous study detected no Bacteroidetes in wild *S. murinus* [9]. Bacteroidetes are common in the mammalian gut, but very few Bacteroidetes are found in insectivorous mammals such as hedgehogs [9, 14]. The low relative abundance of Bacteroidetes in faecal samples of *S. murinus* may be due to its insectivorous diet.

A previous study identified Firmicutes and Bacteroidetes as predominant phyla in human faecal samples [15]. In addition, Lactobacillus constitutes a significant component of the human gut microbiome [16]. We observed a greater similarity between the composition of gut bacteria in humans and *R. norvegicus* than between those of humans and *S. murinus*. This was consistent with the results of throat swab samples and suggests that *R. norvegicus* might be a suitable experimental model for studies related to human oropharyngeal and intestinal function, metabolism and diseases.

*S. murinus* naturally lives in forests and mainly feeds on invertebrates [11]. However, increasingly dense human populations are causing an inevitable reduction and fragmentation of animal habitats, and *S. murinus* is becoming a commensal animal [11]. This provides more opportunities for human–animal contact, and therefore higher rates of bacterial transmission are likely [11]. *R. norvegicus* is considered to be the most commensal synanthropic rodent. This might explain the greater similarity in faecal and throat swab bacterial composition between humans and *R. norvegicus* than between humans and *S. murinus*.

We identified a higher relative abundance of potential pathogens in *S. murinus* than in *R. norvegicus*, in both throat swab and faecal samples (Fig. 10). *Escherichia albertii* is a zoonotic enteropathogen associated with infections among humans and birds [17]. It was detected with high relative abundance (10.22%) in faecal samples of *S. murinus. Clostridium perfringens*, a cause of food poisoning, has also been found with high relative abundance (12.96%) in faecal samples from *S. murinus*. *Lactococcus garvieae* is a known fish pathogen [18]; *Comamonas testosteroni* is an unusual bacteria associated with acute appendicitis [19]; and *Pseudomonas aeruginosa* can cause infections in blood and lungs [20]. These bacteria can be transmitted by contaminated food and water, and they had a greater relative abundance in faecal samples of *S. murinus* than in those of *R. norvegicus*. These observations indicate that *S. murinus* may pose a high potential risk for spreading emerging infectious diseases via water or food contaminated with faeces.

*Fusobacterium russii*, *Prevotella denticola* and *Prevotella melaninogenica* were more common in throat swab samples of *S. murinus* than in those of *R. norvegicus*. These bacteria are associated with bite-wound infections by animals such as cats and dogs [21]. Since *S. murinus* now has more opportunities for human–animal contact, higher rates of pathogen transmission are likely [11]. Increasing attention should therefore be paid to *S. murinus*.

In our study, faecal samples from *R. norvegicus* also contained a variety of bacterial pathogens. *Fusobacterium mortiferum*, an opportunistic pathogen associated with anaerobic sepsis [22], was common in faecal samples from *R. norvegicus* (5.88%). *Streptococcus minor* and *S. aureus* were detected in throat swab samples of *R. norvegicus* with a relative abundance over 1%. *S. minor* causes human wound infections and can be transmitted by dog bites [23], whereas *S. aureus* is a common cause of respiratory infections and food poisoning [24]. Previous research in our laboratory detected MRSA isolates in *R. norvegicus* and *S. murinus* collected in Guangzhou, with a higher detection rate in the former [25]. Similar to *S. murinus*, pathogens detected in throat swab and faecal samples of *R. norvegicus* can cause human diseases by various routes, including bites, saliva and contaminated water and food.

Some severe infectious diseases, such as bartonellosis, leptospirosis, rat-bite fever and plague, are transmitted by rodents. High prevalence of Bartonella has been found in blood samples of *S. murinus* and *R. norvegicus* [26]. However, in our study, none of the faecal or throat swab samples was positive for *Bartonella*. This indicates that *Bartonella* might not be shed in faeces or saliva. Leptospirosis, an important zoonotic infectious disease, used to be prevalent in China, but incidence has gradually decreased in recent years [27]. *Leptospira* has been found in renal samples of *R. norvegicus* in China [28], however, it was not detected in the samples from our study; this could possibly be explained by the sample type. Host animals, such as rats, carry *Leptospira* in their kidneys and shed pathogenic leptospires in their urine [29]. *Streptobacillus moniliformis* and *Spirillum minus,* pathogens of rat-bite fever, naturally colonize the respiratory tracts of rodents [5]. A case of rat-bite fever with *S. moniliformis* was reported in China in 2019, and the patient recalled being bitten by wild rats 1 week prior to the onset of symptoms [30]. In our study, *S. moniliformis* and *S. minus* were not detected in *S. murinus* or *R. norvegicus*. We consider that in Guangdong, China, the transmission of rat-bite fever by *S. murinus* and *R. norvegicus* is unlikely. *Yersinia pestis* (which causes plague disease) is usually found in wild rodents and Asian house shrews and can be transmitted by the bite of infected fleas [3]. In China, the re-emergence of human plague has been reported [31]. After infection, *Y. pestis* spreads to the bloodstream and disseminates to the spleen, liver and other organs [32]. As with *Bartonella* and *Leptospira*, the absence of *Y. pestis* in our study might also be explained by the sample types.

Both *R. norvegicus* and *S. murinus* live and feed in closer proximity to human populations than most other mammals and may serve as potential sources of infectious diseases for humans via cross-species transmission. However, less attention has been paid to *S. murinus* than to *R. norvegicus*. In our study, more sequences from *S. murinus* were annotated as potential pathogens than those from *R. norvegicus*. *S. murinus* might therefore be a more important reservoir of bacterial pathogens than *R. norvegicus*, suggesting that more attention should be paid to *S. murinus* in the prevention of zoonotic diseases in the future.

This study had three main limitations. Firstly, we did not identify the age and sex of the animals. These factors, especially age, might affect the structure of microbial communities within animals. Secondly, the sample size was small. Thirdly, we did not investigate the influence of season. Our findings should be confirmed by more rigorous studies with a larger sample size.

## Conclusion

The results of this study revealed the composition of bacterial communities of the oropharyngeal and gastrointestinal tracts of *R. norvegicus* and *S. murinus*. In general, the bacterial composition of throat swab and faecal samples from *R. norvegicus* were different from those of *S. murinus*. Both *R. norvegicus* and *S. murinus* carried various pathogenic bacteria; however, more sequences from *S. murinus* were annotated as potential human pathogenic bacteria than those from *R. norvegicus*. These results suggest that surveillance of *R. norvegicus* should be continued to prevent outbreaks of zoonotic diseases, and greater attention should be given to *S. murinus* in this regard in the future.

## Methods

### Sample collection

Between May 2015 and April 2016, animals for study were trapped using cages once a month in a residential area of Guangzhou City, China. Trap cages were placed next to human settlements and garbage cans. The trapped animals were anesthetized by the inhalation of 3% diethyl ether. Trained personnel wore filtering facepiece respirators, safety chemical goggles, anti-static uniforms and chemical protective gloves to protect themselves from the diethyl ether. The dosage of diethyl ether was adjusted according to the heart rate, respiratory frequency, corneal reflection and extremity muscle tension of the animal. Throat swab and faecal samples were then collected. Animals were executed via cervical dislocation by trained personnel. Liver tissue samples were collected by intraperitoneal surgery in the laboratory and stored in RNAlater (Invitrogen, CA, USA). Animal species were identified by sequencing the cytochrome B (cytB) gene of liver tissue and morphological identification [33]. Of the animals trapped each month, one individual of each species was randomly selected. Throat swabs and faeces from the selected animals were collected and stored at − 80 °C.

### DNA extraction and amplicon generation

DNA was extracted from throat swab and faecal samples using the QIAamp DNA Microbiome Kit (Qiagen, Germany) and QIAamp DNA Stool Mini Kit (Qiagen), respectively. The V4 hypervariable region of the 16S rRNA gene was amplified with barcoded primers developed in a previous study (515F and 806R) using Phusionign-kh-Fidelity PCR Master Mix (New England Biolabs, UK) [34]. Libraries were generated and sequenced on the Illumina HiSeq 2500 platform.

### Data analyses

Reads were assigned to samples based on their barcode. Raw tags were obtained by overlapping the reads [35]. High-quality clean tags were obtained according to the QIIME (V1.7.0) quality control process [35]. Effective tags were obtained after comparison with the reference database (Gold Database, http://drive5.com/uchime/uchime_download.html) [36].

Sequences with a similarity greater than 97% were assigned to the same OTU. A representative sequence for each OTU was selected for further analyses. Each representative sequence was annotated with taxonomic information using the GreenGene Database (http://greengenes.lbl.gov/cgi-bin/nph-index.cgi) based on the Ribosomal Database Project (RDP) classifier (version 2.2) algorithm [37].

### Ethics statement

The study protocol was approved by the Animal Ethics and Welfare Committee of the School of Public Health, Southern Medical University and adhered to the guidelines for the Rules for the Implementation of Laboratory Animal Medicine (1998) from the Ministry of Health, China. All surgical procedures were performed under anesthesia in efforts to minimize suffering. Endangered or protected species were not involved in this study.

## Supplementary information

**Additional file 1: Figure S1.** Rarefaction curves for the comparison of the microbial communities in different groups of samples. The depth of sequencing was sufficient to reflect bacterial community composition of the samples.

**Additional file 2: Table S1.**The summary of samples collection, grouping and sequencing results.

## Data Availability

The datasets used and analyzed during the current study are available from the corresponding author on reasonable request.

## Abbreviations

*C. perfringens*: *Clostridium perfringens*
*E. albertii*: *Escherichia albertii*
MRSA: Methicillin-resistant *Staphylococcus aureus*
NGS: Next-generation sequencing
NCBI: National Center for Biotechnology Information
OTUs: Operational taxonomic units
PCA: Principal component analysis
PCoA: Principal coordinates analysis
RDP: Ribosomal Database Project
*R. norvegicus*: *Rattus norvegicus*
*S. aureus*: *Staphylococcus aureus*
*S. murinus*: *Suncus murinus*
*S. minor*: *Streptococcus minor*
*S. moniliformis*: *Streptobacillus moniliformis*
*S. minus*: *Spirillum minus*
SRA: Sequence Read Archive
*Y. pestis*: *Yersinia pestis*

## References

[1] Taylor LH, Latham SM, Woolhouse ME (2001). Risk factors for human disease emergence. Philos Trans R Soc Lond Ser B Biol Sci.

[2] Heuser E, Fischer S, Ryll R, Mayer-Scholl A, Hoffmann D, Spahr C, Imholt C, Alfa DM, Frohlich A, Luschow D (2017). Survey for zoonotic pathogens in Norway rat populations from Europe. Pest Manag Sci.

[3] Rahelinirina S, Rajerison M, Telfer S, Savin C, Carniel E, Duplantier JM (2017). The Asian house shrew Suncus murinus as a reservoir and source of human outbreaks of plague in Madagascar. PLoS Negl Trop Dis.

[4] Delahoy MJ, Wodnik B, McAliley L, Penakalapati G, Swarthout J, Freeman MC, Levy K (2018). Pathogens transmitted in animal feces in low- and middle-income countries. Int J Hyg Environ Health.

[5] Hryciw BN, Wright CP, Tan K (2018). Rat bite fever on Vancouver Island: 2010-2016. Can Commun Dis Rep.

[6] Weir GM, MacDonald LD, Rajagopalan R, Sivko GS, Valderas MW, Rayner J, Berger BJ, Sammatur L, Stanford MM (2019). Single dose of DPX-rPA, an enhanced-delivery anthrax vaccine formulation, protects against a lethal Bacillus anthracis spore inhalation challenge. NPJ Vaccines.

[7] Ge J. Carriage and microbiological characteristics of methicillin-resistant Staphylococcus aureus from urban rodent-like animals in Guangzhou and Xiamen: Southern Medical University; 2017. https://kns.cnki.net/kcms/detail/detail.aspx?dbcode=CMFD&dbname=CMFD201801&filename=1017230245.nh&v=OJcedcxxnOP6FgdUd3E1lZ%25mmd2FI0sGw33pf9ITP6enCc6r5h9ZScUV%25mmd2BAmm8UQdZrMmc.

[8] Lee MJ, Byers KA, Donovan CM, Zabek E, Stephen C, Patrick DM, Himsworth CG (2019). Methicillin-resistant Staphylococcus aureus in urban Norway rat (Rattus norvegicus) populations: epidemiology and the impacts of kill-trapping. Zoonoses Public Health.

[9] Shinohara A, Nohara M, Kondo Y, Jogahara T, Nagura-Kato GA, Izawa M, Koshimoto C (2019). Comparison of the gut microbiotas of laboratory and wild Asian house shrews (Suncus murinus) based on cloned 16S rRNA sequences. Exp Anim.

[10] Cong W, Xing J, Feng Y, Wang J, Fu R, Yue B, He Z, Lin L, Yang W, Cheng J (2018). The microbiota in the intestinal and respiratory tracts of naked mole-rats revealed by high-throughput sequencing. BMC Microbiol.

[11] Rahman M, Islam S, Masuduzzaman M, Alam M, Chawdhury MNU, Ferdous J, Islam MN, Hassan MM, Hossain MA, Islam A (2018). Prevalence and diversity of gastrointestinal helminths in free-ranging Asian house shrew (Suncus murinus) in Bangladesh. Vet World.

[12] Gong H, Shi Y, Zhou X, Wu C, Cao P, Xu C, Hou D, Wang Y, Zhou L (2014). Microbiota in the throat and risk factors for laryngeal carcinoma. Appl Environ Microbiol.

[13] Li D, Chen H, Mao B, Yang Q, Zhao J, Gu Z, Zhang H, Chen YQ, Chen W (2017). Microbial biogeography and core microbiota of the rat digestive tract. Sci Rep.

[14] Ley RE, Hamady M, Lozupone C, Turnbaugh PJ, Ramey RR, Bircher JS, Schlegel ML, Tucker TA, Schrenzel MD, Knight R (2008). Evolution of mammals and their gut microbes. Science (New York, NY).

[15] Jandhyala SM, Talukdar R, Subramanyam C, Vuyyuru H, Sasikala M, Nageshwar Reddy D (2015). Role of the normal gut microbiota. World J Gastroenterol.

[16] Goldstein EJ, Tyrrell KL, Citron DM (2015). Lactobacillus species: taxonomic complexity and controversial susceptibilities. Clin Infect Dis.

[17] Murakami K, Maeda-Mitani E, Kimura H, Honda M, Ikeda T, Sugitani W, Konno T, Kawano K, Etoh Y, Sera N (2019). Non-biogroup 1 or 2 strains of the emerging zoonotic pathogen Escherichia albertii, their proposed assignment to biogroup 3, and their commonly detected characteristics. Front Microbiol.

[18] Wang CY, Shie HS, Chen SC, Huang JP, Hsieh IC, Wen MS, Lin FC, Wu D (2007). Lactococcus garvieae infections in humans: possible association with aquaculture outbreaks. Int J Clin Pract.

[19] Bayhan GI, Tanir G, Karaman I, Ozkan S (2013). Comamonas testosteroni: an unusual bacteria associated with acute appendicitis. Balkan Med J.

[20] Berube BJ, Rangel SM, Hauser AR (2016). Pseudomonas aeruginosa: breaking down barriers. Curr Genet.

[21] Alexander CJ, Citron DM, Hunt Gerardo S, Claros MC, Talan D, Goldstein EJ (1997). Characterization of saccharolytic Bacteroides and Prevotella isolates from infected dog and cat bite wounds in humans. J Clin Microbiol.

[22] Prout J, Glymph R (1986). Anaerobic septicemia secondary to Fusobacterium mortiferum. J Natl Med Assoc.

[23] Tre-Hardy M, Saussez T, Yombi JC, Rodriguez-Villalobos H (2016). First case of a dog bite wound infection caused by Streptococcus minor in human. New Microbes New Infect.

[24] Hennekinne JA, De Buyser ML, Dragacci S (2012). Staphylococcus aureus and its food poisoning toxins: characterization and outbreak investigation. FEMS Microbiol Rev.

[25] Ge J, Zhong X, Xiong Y, Qiu M, Huo S, Chen X, Mo Y, Cheng M, Chen Q. Methicillin-resistant Staphylococcus aureus among urban rodents, house shrews, and patients in Guangzhou, southern China. BMC Vet Res. 2019;15(1):260. 10.1186/s12917-019-2012-8 PMC6659301 31345215

[26] Hsieh JW, Tung KC, Chen WC, Lin JW, Chien LJ, Hsu YM, Wang HC, Chomel BB, Chang CC (2010). Epidemiology of Bartonella infection in rodents and shrews in Taiwan. Zoonoses Public Health.

[27] Hu W, Lin X, Yan J (2014). Leptospira and leptospirosis in China. Curr Opin Infect Dis.

[28] Wang ZD, Wang SS, Liu LJ, Yang Y, Li M, Guo TY, Fu YQ, Hou Y, Sun XH, Xu BL (2013). The infection status of Leptospira in rodents on the Heixiazi island of Heilongjiang province, China, in 2011. Zhonghua Yu Fang Yi Xue Za Zhi.

[29] Haake DA, Levett PN (2015). Leptospirosis in humans. Curr Top Microbiol Immunol.

[30] Mai W, Chen S, Chen D, Zhu X, Wang R (2019). One case of rat bite fever caused by Streptobacillus moniliformis. China Trop Med.

[31] Shi L, Yang G, Zhang Z, Xia L, Liang Y, Tan H, He J, Xu J, Song Z, Li W (2018). Reemergence of human plague in Yunnan, China in 2016. PLoS One.

[32] Du Z, Wang X (2016). Pathology and pathogenesis of Yersinia pestis. Adv Exp Med Biol.

[33] Linacre A, Lee JC (2016). Species determination: the role and use of the cytochrome b gene. Methods Mol Biol (Clifton, NJ).

[34] Caporaso JG, Lauber CL, Walters WA, Berg-Lyons D, Huntley J, Fierer N, Owens SM, Betley J, Fraser L, Bauer M (2012). Ultra-high-throughput microbial community analysis on the Illumina HiSeq and MiSeq platforms. ISME J.

[35] Magoc T, Salzberg SL (2011). FLASH: fast length adjustment of short reads to improve genome assemblies. Bioinformatics (Oxford, England).

[36] Haas BJ, Gevers D, Earl AM, Feldgarden M, Ward DV, Giannoukos G, Ciulla D, Tabbaa D, Highlander SK, Sodergren E (2011). Chimeric 16S rRNA sequence formation and detection in Sanger and 454-pyrosequenced PCR amplicons. Genome Res.

[37] Wang Q, Garrity GM, Tiedje JM, Cole JR (2007). Naive Bayesian classifier for rapid assignment of rRNA sequences into the new bacterial taxonomy. Appl Environ Microbiol.

