# Supplementary figures and images for: Composition of gut and oropharynx bacterial communities in Rattus norvegicus and Suncus murinus in China
Source: BMC Vet Res. 2020 Oct 31;16:413. doi: 10.1186/s12917-020-02619-6 (PMC7603701; doi:10.1186/s12917-020-02619-6)

Figure S1

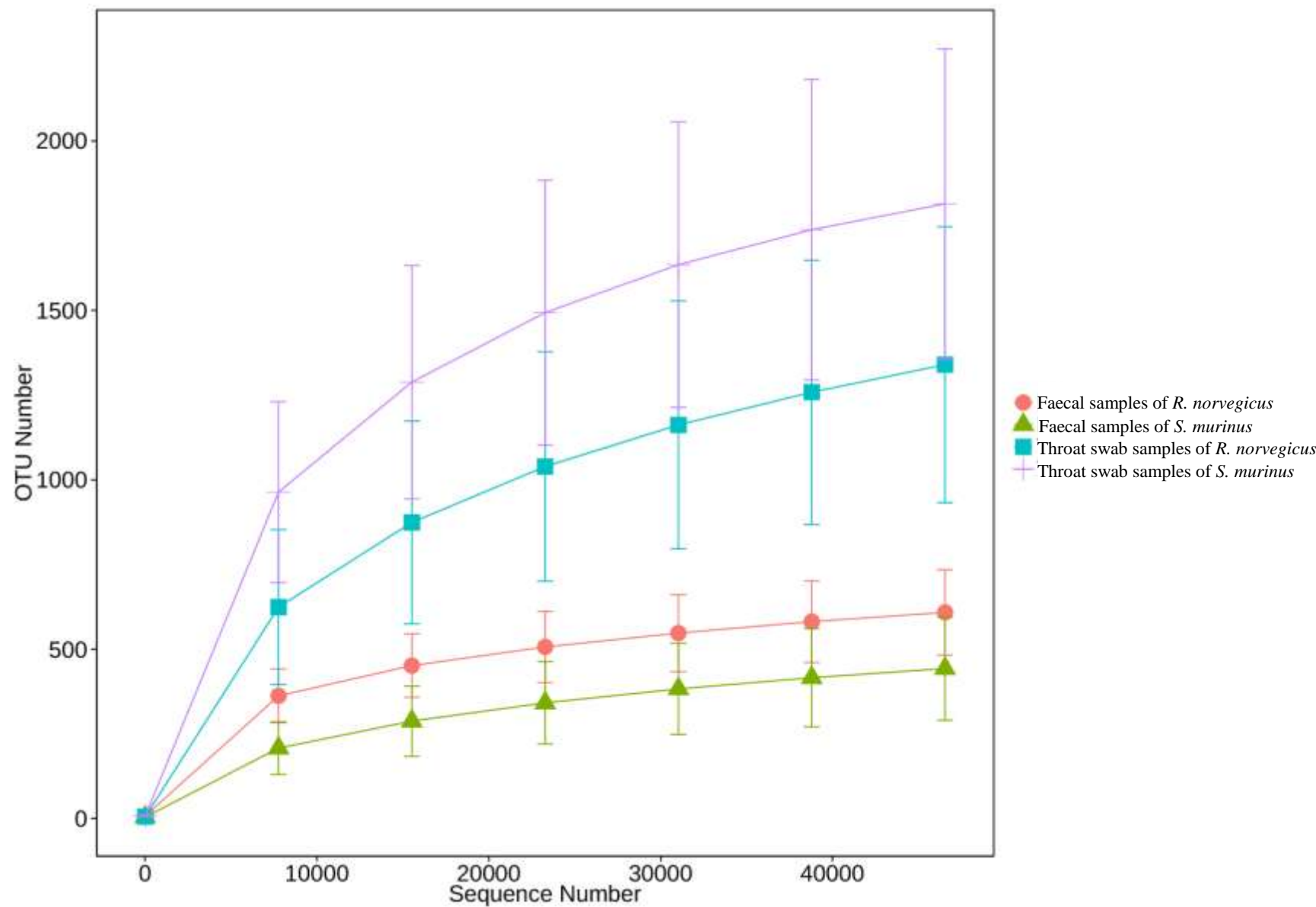

Supplement: Supplementary file 1 — Additional file 1: Figure S1. Rarefaction curves for the comparison of the microbial communities in different groups of samples. The depth of sequencing was sufficient to reflect bacterial community composition of the samples. [file 12917_2020_2619_MOESM1_ESM.pdf]
