# Supplementary material for: Composition of gut and oropharynx bacterial communities in Rattus norvegicus and Suncus murinus in China
Source: BMC Vet Res. 2020 Oct 31;16:413. doi: 10.1186/s12917-020-02619-6 (PMC7603701; doi:10.1186/s12917-020-02619-6)
Supplement: Supplementary file 2 — Additional file 2: Table S1.The summary of samples collection, grouping and sequencing results. [file 12917_2020_2619_MOESM2_ESM.docx]

Table S1.The summary of samples collection, grouping and sequencing results

| Sample type | Sample name | Group | Species | Sampling time | Number of reads | Number of clean tags | Number of effective tags | Number of OTU |
| --- | --- | --- | --- | --- | --- | --- | --- | --- |
| Faecal  samples | R15.5 | F.R | *R. norvegicus* | 2015.05 | 84,242 | 82,828 | 81,165 | 268 |
|  | R15.6 | F.R | *R. norvegicus* | 2015.06 | 96,407 | 93,965 | 91,741 | 599 |
|  | R15.7 | F.R | *R. norvegicus* | 2015.07 | 84,526 | 82,123 | 79,568 | 606 |
|  | R15.8 | F.R | *R. norvegicus* | 2015.08 | 97,272 | 95,068 | 94,116 | 525 |
|  | R15.9 | F.R | *R. norvegicus* | 2015.09 | 86,332 | 84,840 | 83,146 | 559 |
|  | R15.10 | F.R | *R. norvegicus* | 2015.10 | 95,298 | 92,952 | 90,880 | 631 |
|  | R15.11 | F.R | *R. norvegicus* | 2015.11 | 87,186 | 84,238 | 80,939 | 677 |
|  | R15.12 | F.R | *R. norvegicus* | 2015.12 | 91,970 | 89,909 | 86,854 | 622 |
|  | R16.1 | F.R | *R. norvegicus* | 2016.01 | 81,702 | 79,792 | 77,119 | 523 |
|  | R16.2 | F.R | *R. norvegicus* | 2016.02 | 95,942 | 94,212 | 92,856 | 477 |
|  | R16.3 | F.R | *R. norvegicus* | 2016.03 | 96,394 | 94,652 | 92,011 | 466 |
|  | R16.4 | F.R | *R. norvegicus* | 2016.04 | 87,971 | 86,198 | 83,235 | 549 |
|  | S15.5 | F.S | *S. murinus* | 2015.05 | 86,032 | 81,620 | 74,461 | 437 |
|  | S15.6 | F.S | *S. murinus* | 2015.06 | 83,071 | 81,556 | 80,163 | 344 |
|  | S15.7 | F.S | *S. murinus* | 2015.07 | 89,753 | 87,936 | 86,138 | 507 |
|  | S15.8 | F.S | *S. murinus* | 2015.08 | 91,267 | 89,957 | 88,822 | 415 |
|  | S15.9 | F.S | *S. murinus* | 2015.09 | 83,453 | 82,172 | 81,369 | 239 |
|  | S15.10 | F.S | *S. murinus* | 2015.10 | 82,945 | 83,323 | 80,290 | 399 |
|  | S15.11 | F.S | *S. murinus* | 2015.11 | 84,746 | 83,167 | 81,943 | 446 |
|  | S15.12 | F.S | *S. murinus* | 2015.12 | 90,733 | 88,809 | 88,328 | 355 |
|  | S16.1 | F.S | *S. murinus* | 2016.01 | 86,356 | 84,144 | 82,687 | 512 |
|  | S16.2 | F.S | *S. murinus* | 2016.02 | 98,155 | 96,502 | 95,085 | 234 |
|  | S16.3 | F.S | *S. murinus* | 2016.03 | 98,787 | 96,800 | 95,082 | 562 |
|  | S16.4 | F.S | *S. murinus* | 2016.04 | 98,984 | 97,213 | 95,398 | 269 |
|  | T.R15.5 | T.R | *R. norvegicus* | 2015.05 | 85,514 | 82,053 | 76,927 | 1476 |
|  | T.R15.6 | T.R | *R. norvegicus* | 2015.06 | 94,390 | 81,401 | 74,699 | 697 |
| Throat swab  samples | T.R15.7 | T.R | *R. norvegicus* | 2015.07 | 88,466 | 81,825 | 79,147 | 1259 |
|  | T.R15.8 | T.R | *R. norvegicus* | 2015.08 | 85,732 | 75,129 | 72,810 | 1229 |
|  | T.R15.9 | T.R | *R. norvegicus* | 2015.09 | 83,778 | 73,294 | 71,646 | 550 |
|  | T.R15.10 | T.R | *R. norvegicus* | 2015.10 | 88,462 | 82,078 | 78,954 | 1013 |
|  | T.R15.11 | T.R | *R. norvegicus* | 2015.11 | 84,033 | 81,339 | 78,278 | 1026 |
|  | T.R15.12 | T.R | *R. norvegicus* | 2015.12 | 84,871 | 79,478 | 77,878 | 1062 |
|  | T.R16.1 | T.R | *R. norvegicus* | 2016.01 | 94,045 | 84,285 | 81,065 | 945 |
|  | T.R16.2 | T.R | *R. norvegicus* | 2016.02 | 81,973 | 74,299 | 72,210 | 937 |
|  | T.R16.3 | T.R | *R. norvegicus* | 2016.03 | 89,070 | 85,830 | 81,817 | 812 |
|  | T.R16.4 | T.R | *R. norvegicus* | 2016.04 | 96,539 | 84,099 | 81,087 | 1429 |
|  | T.S15.5 | T.S  T.S  T.S  T.S  T.S  T.S  T.S  T.S  T.S  T.S  T.S  T.S | *S. murinus* | 2015.05 | 81,426 | 74,361 | 70,718 | 1681 |
|  | T.S15.6 |  | *S. murinus* | 2015.06 | 78,652 | 68,075 | 62,673 | 1392 |
|  | T.S15.7 |  | *S. murinus* | 2015.07 | 78,597 | 67,999 | 59,149 | 1455 |
|  | T.S15.8 |  | *S. murinus* | 2015.08 | 77,643 | 68,089 | 62,467 | 1549 |
|  | T.S15.9 |  | *S. murinus* | 2015.09 | 94,741 | 90,935 | 87,326 | 525 |
|  | T.S15.10 |  | *S. murinus* | 2015.10 | 84,184 | 81,423 | 70,185 | 1118 |
|  | T.S15.11 |  | *S. murinus* | 2015.11 | 95,987 | 87,552 | 80,265 | 1398 |
|  | T.S15.12 |  | *S. murinus* | 2015.12 | 64,625 | 54,999 | 50,320 | 1509 |
|  | T.S16.1 |  | *S. murinus* | 2016.01 | 66,424 | 55,800 | 51,706 | 1117 |
|  | T.S16.2 |  | *S. murinus* | 2016.02 | 72,782 | 58,656 | 53,740 | 1435 |
|  | T.S16.3 |  | *S. murinus* | 2016.03 | 83,440 | 74,468 | 68,029 | 926 |
|  | T.S16.4 |  | *S. murinus* | 2016.04 | 82,172 | 74,055 | 69,499 | 1735 |

T.S: Throat swab samples from *Suncus murinus*; T.R: Throat swab samples from *Rattus norvegicus*; F.S: Faecal samples from *Suncus murinus*; F.R: Faecal samples from *Rattus norvegicus*; OTU, operational taxonomic units.
